# Do Variations in Frontal Recess Anatomy Predispose to Mucocele Formation?

**DOI:** 10.1055/s-0044-1788002

**Published:** 2025-01-22

**Authors:** Lalee Varghese, Rakesh R. Bright, Aditya V. A. Gunturi, Grace Rebekah, Regi Kurien

**Affiliations:** 1 Department of Otorhinolaryngology, Christian Medical College, Vellore, Tamil Nadu, India; 2 Department of Radiology, Christian Medical College, Vellore, Tamil Nadu, India; 3 Department of Biostatistics, Christian Medical College, Vellore, Tamil Nadu, India

**Keywords:** paranasal sinus, mucocele, frontal cell, frontal sinus

## Abstract

**Introduction** Mucoceles are benign expansile cystic lesions commonly seen in the frontoethmoidal region.

**Objective** To see if the distribution of frontal air cells predisposes to mucocele formation.

**Methods** Retrospective review of all cases of paranasal sinus mucocele from 2011 to 2021. Data on demographics, history of surgery or trauma, clinical features, radiological findings, and outcome were collected and analyzed.

**Results** Of the 28 cases, 19 (67.9%) were male and 9 (32.1%), female, with a mean age of 40.75 years. Mucocele was unilateral in 26 (92.9%) patients. Twenty patients (71.43%) presented with primary mucocele. The distribution of mucocele was frontal and frontoethmoidal in 8 (28.6%) patients each, maxillary in 6 (21.4%), and ethmoid and sphenoid sinus in 3 (10.7%) patients each. Sixteen (57.1%) patients had frontal sinus involvement. At presentation, 13 (46.4%) patients had nasal symptoms, 17 (60.7%) had orbital symptoms, while 16 (57.1%) had headache. Pain (12; 70.59%) was the predominant orbital symptom, followed by proptosis and diplopia (8; 47.06%). The most common sites of bony erosions were along the frontal sinus floor (14; 50%), followed by lamina papyracea (13; 46.43%), and frontal sinus anterior wall (10; 35.71%). The agger nasi and suprabullar cells were the most common frontal cells encountered in mucoceles involving the frontal sinus, with no significant difference in frontal cell distribution between involved and uninvolved sides. The frontal cell distribution was similar in mucoceles with and without frontal sinus involvement too.

**Conclusion** Though frontal and frontoethmoidal mucoceles were the most encountered, the type and distribution of frontal cells did not predispose to mucocele formation.

## Introduction

Mucoceles of the paranasal sinuses are benign locally expansile epithelial lined cystic lesions filled with mucoid or mucopurulent secretions. These lesions are usually lined by pseudostratified columnar epithelium. Some cases may be associated with areas of squamous epithelium, inflammatory cell infiltration, bone resorption, and new bone formation. 1 Though described in the 1700s, the exact etiological mechanisms are still debatable.

Mucoceles are classified into primary and secondary. Primary mucoceles occur de novo, while secondary mucoceles arise from previous injuries, such as sinus surgery or trauma. Expansion of mucocele is a culmination of various mechanical stress induced remodeling and biochemical osteoclastic bone resorption secondary to sinus obstruction and chronic inflammation. 2 Close proximity to orbit and the relatively thin lamina papyracea make ophthalmic manifestations a common presentation in patients with frontoethmoidal mucoceles. Endoscopic marsupialization, which is a minimally invasive, reliable approach, forms the mainstay of treatment of mucoceles, while external approaches are recommended only for a few select indications. 3

Although relatively commonly seen in the frontoethmoidal region, a review of the literature showed no definite reason contributing to the higher incidence of mucoceles in this region compared with other sinuses. We hypothesize that this could be attributed to the relatively complex anatomy of the frontal recess and the adjoining ethmoidal air cells. Moreover, minor trauma and secondary scarring could further attenuate the already narrow drainage pathway. Therefore, the present study aimed to assess the distribution of cells in the frontoethmoidal region and to correlate its predisposition in mucocele development.

## Methods

This was a retrospective analysis of all patients operated for paranasal sinus mucocele at a tertiary care teaching hospital in India from 2011 to 2021. After obtaining Institutional Review Board and Ethics Committee approval (IRB No.14401), the following data were obtained from patient medical records: age, gender, medical comorbidities, history of surgery or trauma, clinical features, extent of sinonasal involvement, radiological findings, and outcome.

### Statistical Analysis

All descriptive statistics were reported as frequency and percentages for categorical variables and mean and standard deviation for continuous variables. Association between frontal cell types and frontal mucocele involved and uninvolved sides was reported using the Chi-squared or Fisher exact test. A *p* -value < 0.05 was considered as statistically significant. All statistical analysis was done using the IBM SPSS Statistics for Windows, version 21.0 (IBM Corp., Armonk, NY, USA).

## Results

During the study period, there were 28 cases of paranasal sinus mucoceles of which 19 (67.9%) were male and 9 (32.1%), female. The patients' ages ranged from 20 to 66 years, with a mean age of 40.75. A vast majority of the patients (26; 92.9%) presented with this condition for the first time. Only two had already been operated on before and came with recurrent disease. Mucocele was unilateral in 26 (92.9%) cases, with right side involvement in 14 patients and left side in 12. Two (7.1%) patients had bilateral disease.

Twenty patients (71.43%) presented with primary disease. Mucocele was secondary to previous injury in 8 cases. Six patients (21.43%) had history of a nasal surgery and 2 (7.1%) had a history of trauma ( Table 1 ).

**Table 1:** General characteristics

	Number	Percentage
**Gender**		
Male	19	67.9
Female	9	32.1
**Laterality**		
Unilateral	26	92.9
Right	14	50
Left	12	42.9
Bilateral	2	7.1
**Presentation**		
Primary	20	71.4
Secondary	8	28.6
Prior trauma	2	7.1
Prior surgery (primary condition)	6	21.4
*FESS (SNP)*	*3*	*10.7*
*FESS+ Caldwell Luc (SNP)*	*1*	*3.6*
*Lynch Howarth approach (Mucocele)*	*1*	*3.6*
*Caldwell Luc surgery (Maxillary polyp)*	*1*	*3.6*
**Site of origin of mucocele**		
Frontal	8	28.6
Ethmoidal	3	10.7
Frontoethmoidal	8	28.6
Maxillary	6	21.4
Sphenoidal	3	10.7
**Extraparanasal sinus extension**		
Intraorbital	18	64.29
Intracranial	2	7.14

Abbreviations: FESS, Functional endoscopic sinus surgery; SNP, Sinonasal polyposis.

The most common sites of origin of mucocele were frontal and frontoethmoidal (8; 28.6% patients each) followed by maxillary in 6 (21.4%) patients. Three (10.7%) patients each had ethmoid and sphenoid sinus mucocele. Altogether, the frontal sinus was involved in most cases (16; 57.1%), followed by the ethmoid sinus in 11 (39.3%) patients. Among the 6 patients with maxillary mucocele, 2 (33.33%) had previously undergone Caldwell Luc surgery. The rest of the patients with prior surgery or trauma developed either frontal or frontoethmoidal mucocele.

The most common presenting complaint was headache (16; 57.1%). At presentation, 13 (46.4%) patients had nasal symptoms and 17 (60.7%) had orbital symptoms ( Table 2 ). Among the nasal symptoms, the most reported was nasal obstruction (11; 84.6% patients), followed by nasal discharge (4; 30.77%) and epistaxis (2; 14.38%). Pain (12; 70.59%) was the predominant orbital symptom, followed by proptosis and diplopia (8; 47.06%). Two patients had reduced vision at presentation.

**Table 2:** Clinical presentation

Presenting complaints	Number	Percentage
***Nasal symptoms***	**13**	**46.4**
Nasal obstruction	11	84.6
Nasal discharge	4	30.77
Epistaxis	2	14.38
Blood-stained discharge	1	7.69
Loss of smell	1	7.69
***Orbital symptoms***	**17**	**60.7**
Orbital pain	12	70.59
Proptosis	8	47.06
Diplopia	8	47.06
Epiphora	5	29.41
Reduced vision	2	11.76
***Others***		
Headache	16	57.1
Facial pain	2	7.1
Forehead swelling	5	17.9
Cheek swelling	4	14.3
Palatal bulge	1	3.6

On clinical examination, 10 (35.7%) patients had swelling in the medial canthus, and 1 patient each showed nasal external framework widening and palatal bulge. The eye was pushed down in 7 (25%) patients, and laterally in 6 (21.4%). Extraocular movements were affected in 10 (35.71%) patients, with the movement restricted being abduction in 2, adduction in 3, depression in 1, and elevation in 9 patients. In one patient, all four movements were impaired.

**Fig. 1:**
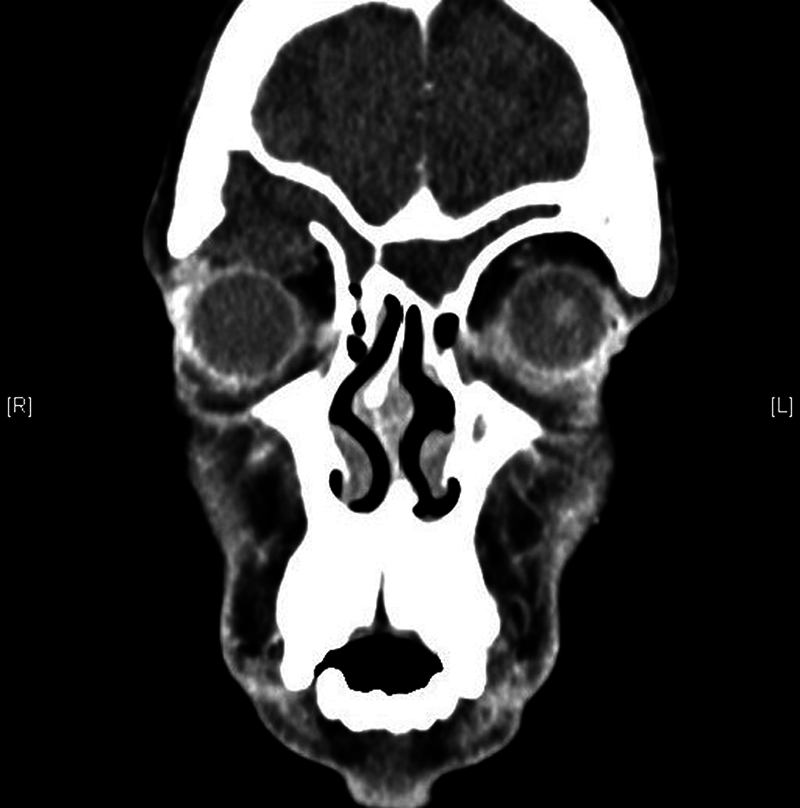
Right frontal sinus mucocele with erosion of floor and intraorbital extension.

Diagnostic nasal endoscopy revealed discharge in 8 (28.6%) patients and polyp in 6 (21.4%). Table 3 depicts the various sites where bony erosions were seen on computed tomography (CT) scan. Most patients showed erosions along the frontal sinus floor (14; 50%) followed by lamina papyracea (13; 46.43%) and frontal sinus anterior wall (10; 35.71%). A total of 75% of patients with frontal mucocele and all patients with frontoethmoidal mucocele had erosion over the floor of the frontal sinus ( Fig. 1 ). Among the frontoethmoidal mucocele patients, 87.5% showed frontal anterior wall erosion, and 75% showed erosion of frontal posterior wall. All patients with frontoethmoidal and ethmoidal mucoceles also showed associated lamina papyracea dehiscence.

**Table 3:** Sites of bony erosions

	Frontal mucocele ( *n* = 8)		Frontoethmoidal mucocele ( *n* = 8)		Ethmoidal mucocele ( *n* = 3)		Maxillary mucocele ( *n* = 6)		Sphenoid mucocele ( *n* = 3)		Total ( *n* = 28)	
	n	%	n	%	n	%	n	%	n	%	n	%
**Frontal sinus**												
Anterior wall	3	37.5	7	87.5	0	0	0	0	0	0	10	35.71
Posterior wall	2	25	6	75	0	0	0	0	0	0	8	28.57
Floor	6	75	8	100	0	0	0	0	0	0	14	50
**Ethmoid sinus**												
Cribriform plate	0	0	1	12.5	0	0	0	0	0	0	1	3.57
Lamina papyracea	1	12.5	8	100	3	100	1	16.67	0	0	13	46.43
Fovea ethmoidalis	0	0	2	25	1	33.33	0	0	0	0	3	10.71
**Maxillary sinus**												
Anterior wall	0	0	0	0	0	0	4	66.67	0	0	4	14.29
Posterior wall	0	0	0	0	0	0	3	50	0	0	3	10.71
Medial wall	0	0	0	0	0	0	4	66.67	0	0	4	14.29
Roof	0	0	0	0	0	0	2	33.33	0	0	2	7.14
Floor	0	0	0	0	0	0	1	16.67	0	0	1	3.57
**Sphenoid sinus**	0	0	0	0	0	0	0	0	2	66.67	2	7.14
Intraorbital extension	6	75	8	100	2	66.67	2	33.33	0	0	18	64.29
Intracranial extension	1	12.5	1	12.5	0	0	0	0	0	0	2	7.14

Table 4 depicts the frontal cell distribution based on the international frontal sinus anatomy classification. Agger nasi and suprabullar cells were the most common frontal cells encountered in mucoceles involving frontal sinus. This was true for both involved and uninvolved sides. However, neither the involved nor the uninvolved side showed any statistically significant difference in the distribution of any of the frontal cells. Also, there was no significant difference in frontal cell distribution between mucoceles with and without frontal sinus involvement.

**Table 4:** Frontal cells in mucoceles

Frontal cell type	Frontal and frontoethmoidal mucoceles involved vs uninvolved sides			Frontal and frontoethmoidal mucoceles involved side vs other mucoceles	
	Involved side ( *n* = 15)	Uninvolved side ( *n* = 13)	*p* -value	Other mucoceles ( *n* = 14)	*p* -value
Agger nasi	13 (86.67%)	6 (46.15%)	0.06	11 (78.57%)	0.69
Supra agger nasi	4 (26.67%)	4 (30.77%)	0.09	4 (28.57%)	0.84
Supra agger frontal	1 (6.67%)	1 (7.69%)	−	1 (7.14%)	–
Suprabullar	5 (33.33%)	6 (46.15%)	0.67	6 (42.86%)	0.58
Suprabullar frontal	4 (26.67%)	3 (23.77%)	0.93	1 (7.14%)	0.69
Supraorbital ethmoid	3 (20%)	0	−	0	
Medial frontal	1 (6.67%)	0	−	0	

All patients underwent endoscopic marsupialization, 16 of whom had a follow-up of more than 3 months, with none requiring revision surgery for mucocele. However, three patients underwent a second surgery for a coexistent condition (extranasopharyngeal angiofibroma, frontal sinus cholesteatoma, and recurrent eosinophilic mucinous rhinosinusitis).

## Discussion

Mucoceles are benign expansile cystic lesions involving the paranasal sinuses. In the present study, the majority of the mucoceles were unilateral, primary in origin, with 57% showing frontal sinus involvement. However, there was no significant difference in frontal cell distribution between the involved and uninvolved sides.

Though a similar incidence of mucocele has been described in both sexes, 4 almost ⅔ of the patients in our cohort were male. A slight male predominance has also been described by Bouatay et al.. 5 The maximum incidence is reported during the third and fourth decades of life. 4 5 In our cohort, even though the patients' age ranged from 20 to 66 years, with a mean of 40.75, the highest numbers were seen in the 5 ^th^ decade (9 patients), followed by the 3 ^rd^ decade (7 patients).

Based on their origin, mucoceles are classified as primary and secondary. 2 Primary mucoceles occur without any previous injury and are thought to result from mucus drainage block due to inflammation, secretory duct obstruction, mucous gland cystic dilation, or cystic degeneration of polyps. 4 6 A vast majority of the study patients had primary disease and did not show any side predilection. This is contrary to the observation that primary mucoceles are less common and that mucoceles are secondary in 60 to 90% of cases. 7 Secondary mucoceles occur following prior sinus surgery, intranasal trauma, or external facial trauma. Six (21.43%) patients in the cohort had a history of surgery, and 2 reported history of trauma. Even though generally bilateral mucoceles are considered uncommon, the present cohort had 2 patients, accounting for 7.1% cases. Of these, although one patient had undergone FESS previously, the other had no predisposing factors.

Regardless of the underlying cause of mucocele, continued mucus production and accumulation within the obstructed sinus leads to a progressively enlarging mucoperiosteum-lined cyst-like structure. Increased pressure within the mucocele triggers a stress-induced bony remodeling. Also, cytokines, collagenases, and prostaglandins released due to chronic inflammation stimulate osteoclastic bone resorption. These mechanical and biochemical factors lead to the bony changes seen with mucocele expansion. 8

The distribution of mucocele in our series was like that described in literature, with the majority being frontal or frontoethmoidal mucoceles. 2 Altogether, frontal and ethmoidal sinus involvement was seen in 16 (57.1%) and 11 (39.3%) patients, respectively. This can probably be attributed to the complex anatomy of the drainage pathway of these sinuses, especially the frontal sinus. 4 However, 21.4% patients had maxillary sinus mucocele, which is much higher than the reported rate of less than 10%. Ethmoid and sphenoid sinus mucoceles, although rare, representing only 1% of all paranasal sinus mucoceles, were also more prevalent in our series.

Mucoceles are known to remain asymptomatic for a long time. Once they progressively enlarge, depending on the site and size, they may present with rhinological, ophthalmological or neurological symptoms due to compression of the contiguous structures. 5 It has been reported that 70% of patients with ethmoid or sphenoid mucoceles present to ophthalmologists. 6 Most patients in the series presented with orbital symptoms or headache. The most common eye symptom was pain, followed by proptosis and diplopia. Nasal symptoms were seen only in 46.4% of cases, with nasal obstruction being the most predominant one. Other presentations included forehead swelling, cheek swelling, palatal bulge, and facial pain.

Frontoethmoidal mucoceles are known to present with proptosis, inferior displacement of eye globe or diplopia. There may be associated swelling in the medial aspect of roof of orbit, raised intraocular pressure or choroidal fold. 9 Occasionally, they produce forehead swelling. Maxillary mucoceles on medial expansion push the medial wall of the sinus causing nasal obstruction. Superior extension causes displacement of orbital contents and visual changes, inferior extension results in palatal bulge and loosening of teeth, and anterior extension cause painless cheek swelling. 8 In our series, 35.71% of the patients had restriction of extraocular movements with the most affected being elevation (9 patients). A quarter of the patients had the globe displaced down, and in 21% it was pushed laterally. These could be explained by the predominance of frontal sinus involvement in the study subjects. Visual disturbances are rare occurrences with mucoceles, and we encountered two such patients. Two postulated mechanisms are ischemia and venous congestion due to pressure of expanding mucocele on dehiscent or eroded optic nerve canal and inflammation due to spread of infection from mucoceles. Visual disturbances are more common in rapidly expanding mucoceles.

Computed tomography (CT) of paranasal sinuses is the radiological investigation of choice. Mucoceles appear as homogeneous isodense lesions that do not enhance with contrast unless infected. The lining mucosa may often show a fine peripheral enhancement. Magnetic resonance imaging (MRI) is indicated in cases with suspected intracranial or intraorbital extension. On MRI, mucoceles show variable signal intensity in T1- and T2-weighted images, depending on the fluid and protein content or the degree of dehydration. 4 Compared with MRI, the advantage of CT is its ability to show bony detail, which helps to delineate bony expansion, erosions, and intraorbital or intracranial extensions. In this series, the most common areas of bony erosions were the frontal sinus floor followed by lamina papyracea.

The intricate and complex anatomy of the frontal drainage pathway is thought to be the reason for preponderance of mucoceles in these sinuses. In the present study, agger nasi and suprabullar cells were the most common frontal cells encountered in mucoceles involving the frontal sinus. There was no statistically significant difference in the distribution of any of the frontal cells between the involved and uninvolved sides. The presence of different frontal cells in mucoceles with and without frontal sinus involvement was also comparable. Therefore, no particular type of frontal cell was found to be predisposing to mucocele formation.

Endoscopic marsupialization has evolved as the treatment of choice for mucoceles, replacing the previously described external approaches. The obstructed sinus drainage pathway is opened, and changes like cicatrization, adhesion, and bone hyperplasia, if any, are addressed. Endoscopic marsupialization has the advantages of being minimally invasive, avoiding external incision, allowing good visualization, having a smaller chance of complications, and requiring briefer hospital stay. It also enables sinus function to revert to normal, with restored mucociliary clearance, and helps the surgeon to endoscopically examine the sinus on follow-up. However, endoscopic surgery alone may not be sufficient in all cases, and external approaches are still indicated in specific situations, such as laterally placed frontal mucocele, hypertrophic bone occluding frontonasal recess, and compartmentalized maxillary mucocele. 4 10

## Conclusion

Most mucoceles are unilateral and primary in origin, with 57% being frontal and frontoethmoidal. Despite the highly variable frontal recess anatomy, the type and distribution of frontal cells did not appear to predispose to mucocele formation.
